# Nursing interventions and multidomain physiological trajectories in ARDS: a retrospective cohort study

**DOI:** 10.3389/fmed.2026.1764005

**Published:** 2026-03-06

**Authors:** Quanxiu Tang, Kunping Cui, Jing Zhou, Yue Ruan, Xia Li

**Affiliations:** 1 Department of Critical Care Medicine, West China Hospital, Sichuan University, Chengdu, Sichuan, China; 2 West China School of Nursing, Sichuan University, Chengdu, Sichuan, China; 3 Center of Infectious Diseases, West China Hospital of Sichuan University, Chengdu, China

**Keywords:** acute respiratory distress syndrome, enteral nutrition, nursing care, prone positioning, trajectory analysis

## Abstract

**Background:**

Nursing interventions play a key role in managing acute respiratory distress syndrome (ARDS), but their relationship with the dynamic physiological changes that occur during treatment is not well described.

**Methods:**

In a retrospective cohort of 1,716 ARDS patients from the West China Hospital Big Data Platform (2012–2025), four nursing interventions, airway suctioning, oral care, prone positioning, and early enteral nutrition were examined. Trajectories of SpO₂, C-reactive protein (CRP), lactate, and creatinine were derived using latent-class mixed-effects models. Associations between interventions and trajectory membership were assessed through multinomial logistic regression.

**Results:**

Three distinct trajectories were identified for each biomarker including oxygenation, inflammation, metabolism, and renal function. Early prone positioning initiated within 24 h was associated with lower odds of unfavorable oxygenation trajectories (SpO₂ trajectory 2: OR 0.08, 95% CI 0.01–0.59) and high-CRP trajectories (OR 0.18, 95% CI 0.04–0.86). Delayed prone positioning (>72 h) was associated with higher odds of both the stable-low lactate trajectory (trajectory 2: OR 5.17, 95% CI 1.21–22.06) and the U-shaped lactate trajectory (trajectory 3: OR 5.07, 95% CI 1.09–23.57). EEN was consistently linked to reduced odds of high-CRP trajectories (OR 0.31–0.45 across models). Airway suctioning was linked to more favorable oxygenation trajectories, while oral care was more common among patients with unstable oxygenation. Patients in unstable oxygenation trajectories had higher 28-day mortality, while the stable lactate trajectory showed a protective association.

**Conclusion:**

Distinct physiological trajectories characterize the early course of ARDS. Early and standardized nursing interventions, particularly prone positioning and enteral nutrition, were associated with more favorable recovery profiles.

## Introduction

Acute respiratory distress syndrome (ARDS) remains a major cause of morbidity and mortality in critical care worldwide. Despite progress in lung-protective ventilation, extracorporeal support, and infection control, the hospital mortality of moderate to severe ARDS continues to range between 30 and 45% across large international cohorts (1, 2). The syndrome is characterized by severe hypoxemia, diffuse alveolar damage, and complex systemic inflammation, and it often evolves into multi-organ dysfunction. Beyond mechanical ventilation, high-quality nursing care plays a decisive role in the treatment and recovery of critically ill patients (3). However, the physiological mechanisms through which specific nursing interventions are associated with outcomes in ARDS have not been comprehensively investigated (4, 5).

Prone positioning is the most established nursing intervention shown to improve survival in ARDS. The PROSEVA randomized trial demonstrated a significant reduction in 28-day mortality (16.0% vs. 32.8%) among patients with severe ARDS who received early and prolonged prone positioning (6). Subsequent meta-analyses and real-world studies have consistently confirmed this survival benefit, further emphasizing the critical importance of early initiation and adequate duration (7–9). Despite robust evidence of benefit, there remains substantial heterogeneity in individual responses, and the underlying physiological pathways linking prone positioning to improved outcomes are not fully understood.

Early enteral nutrition (EEN) and meticulous airway or oral care are other fundamental components of ARDS management (10, 11). Early initiation of enteral feeding in critically ill patients has been associated with reduced infectious complications and shorter ICU stays (10, 12). Similarly, structured airway suctioning and oral hygiene protocols have been linked to lower rates of ventilator-associated pneumonia and systemic infection (13, 14). Although these interventions are widely implemented, previous research has primarily focused on binary outcomes such as mortality, ventilator-free days, or pneumonia incidence. Far less attention has been given to how these nursing practices relate to the trajectory of key physiological indicators during hospitalization.

ARDS is increasingly recognized as a multisystem disorder involving pulmonary, circulatory, inflammatory, and renal dysfunction (15, 16). Monitoring serial changes in representative biomarkers provides an opportunity to capture the course of organ recovery. Peripheral oxygen saturation (SpO₂) reflects gas exchange efficiency, while arterial lactate indicates the adequacy of tissue perfusion and global metabolic stress. C-reactive protein (CRP) mirrors the intensity of systemic inflammation, and serum creatinine represents renal filtration capacity and the presence of acute kidney injury. Persistent elevation or delayed clearance of these markers has been associated with adverse outcomes in critically ill patients. In ARDS, high lactate levels and slow clearance predict increased mortality (17); elevated CRP reflects an ongoing inflammatory burden (18, 19); and renal dysfunction frequently coexists, amplifying mortality risk (20). These indicators may therefore serve as objective windows into the biological response to different nursing interventions. Unlike single-timepoint biomarkers, trajectories capture dynamic physiological responses that may be associated with nursing interventions.

Existing literature has rarely integrated these biomarkers into a trajectory-based framework to describe the physiological associations of nursing care over time. Most prior investigations have focused on the trajectories of ventilatory parameters, such as positive end-expiratory pressure (PEEP), or on fluid balance trends, without considering the role of specific nursing interventions (21, 22). Establishing a biomarker-based trajectory framework may offer a deeper understanding of patient heterogeneity by identifying distinct patterns of improvement or deterioration that precede clinical outcomes. Linking nursing interventions to temporal changes in oxygenation, perfusion, inflammation, and renal function could therefore provide insights that move beyond static associations and help elucidate the biological pathways underlying recovery.

The present study was designed to address these gaps. First, we sought to characterize the longitudinal trajectories of key clinical biomarkers, including SpO₂, lactate, CRP, and creatinine, in patients with ARDS during their ICU stay. Second, we examined how specific nursing interventions, such as prone positioning, EEN, airway suctioning, and oral care, are associated with the evolution of these biomarker trajectories. Third, we explored the association between different trajectory patterns and in-hospital mortality. This study integrates multi-domain physiological data with detailed nursing records to elucidate the association between nursing care, organ recovery, and patient survival in ARDS. Ultimately, these findings aim to inform evidence-based nursing decisions in daily ICU practice.

## Methods

### Data source

This retrospective cohort study utilized data from the Big Data Platform of West China Hospital, Sichuan University, which integrates longitudinal electronic health record data from the West China Hospital system and affiliated medical alliance hospitals across western China (23). The platform contains standardized clinical information extracted from hospital information systems, laboratory information systems, ICU databases, and nursing records, covering over 10 million patient encounters. Data extraction and integration follow uniform data governance, quality control, and security protocols as previously described (23). The reliability and data governance standards of this platform have been rigorously validated and successfully applied in multiple high-quality clinical investigations, including the West-China Hospital Alliance Longitudinal Epidemiology Wellness (WHALE) study (24) and studies identifying prognostic biomarkers in complex comorbidities (25). To ensure data quality for the current longitudinal analysis, the platform employs a standardized governance system incorporating data lineage-based quality control models. We implemented rigorous range checks to exclude physiologically impossible outliers and standardized all laboratory units and reference ranges to ensure consistency across the study period. Furthermore, we cross-referenced timestamps between the Laboratory Information System and nursing records to strictly align all measurements relative to the time of ICU admission. Importantly, West China Hospital is the largest tertiary referral medical center in western China and serves as a national hub for advanced critical care, attracting a high proportion of severe and complex cases from Sichuan Province and neighboring regions. This study was approved by the Ethics Committee of West China Hospital, Sichuan University, with a waiver of informed consent (No. 2025-907).

### Study population

**Figure 1:**
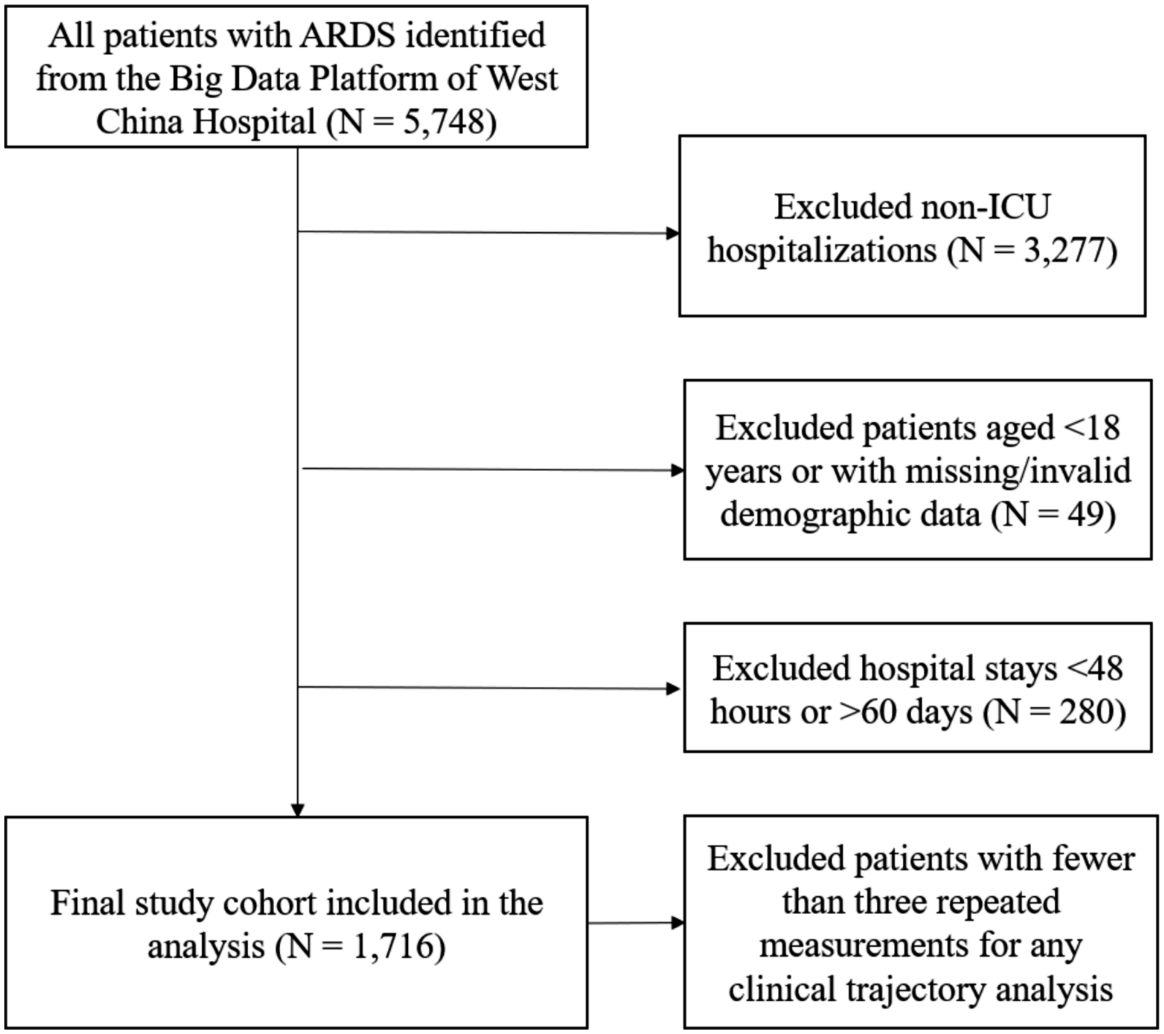
Flowchart of patient inclusion.

The study population comprised critically ill adult patients diagnosed with ARDS who required invasive mechanical ventilation. Eligible cases were identified from the West China Hospital Big Data Platform between January 2012 and July 2025 based on physician documentation and ICD-10 code J80.x. To ensure diagnostic accuracy, all cases were verified to meet the Berlin definition (26), with the timing of diagnosis determined by the first concurrent documentation of a PaO_2_/FiO_2_ ratio of 300 mmHg or less and bilateral opacities on chest imaging while the patient was receiving invasive mechanical ventilation. Patients were excluded if they met any of the following criteria: (1) non-ICU hospitalizations; (2) age < 18 years; (3) missing essential demographic or clinical information such as sex, age, or admission and discharge dates; (4) ICU length of stay shorter than 48 h or longer than 60 days; or (5) incomplete longitudinal biomarker data. For individuals with multiple ICU admissions during the study period, only the first qualifying ICU stay was included in the analysis. The flow of patient inclusion is presented in Figure 1.

### Variables and measurements

Demographic and clinical characteristics were extracted from the electronic medical records, including age, sex, body mass index (BMI), and major comorbidities such as hypertension, diabetes, coronary heart disease (CHD), and chronic kidney disease (CKD). Nursing intervention variables of interest included prone positioning, EEN, airway suctioning, and oral care. Prone positioning was defined as any documented use of prone ventilation within the first 7 days after ICU admission. To further investigate the association of timing, prone positioning was categorized as initiated ≤24 h, 24–48 h, 48–72 h, or >72 h after ICU admission, according to physician and nursing records. The cutoff for early initiation (≤24 h) was selected to align with the protocol of the landmark PROSEVA trial (6) and is supported by recent ESICM guidelines recommending prompt intervention for moderate-to-severe ARDS (27). Biologically, this window corresponds to the early exudative phase of ARDS, characterized by high alveolar recruitability. In contrast, initiation beyond 72 h represents a delayed phase where lung consolidations may become less responsive to recruitment maneuvers as the lung transitions to the fibroproliferative phase (16, 28). The intermediate intervals (24–48 h and 48–72 h) were specified to examine the potential graded attenuation of physiological response during the transition between these two distinct biological phases. As a real-world observational study, the specific implementation protocols were determined by clinician judgment and patient tolerance rather than a standardized research mandate. EEN was defined as the initiation of enteral feeding within 48 h of ICU admission, in accordance with established critical care nutrition guidelines (12). Airway suctioning referred to nursing procedures performed to maintain airway patency and prevent secretion retention in mechanically ventilated patients (29). Oral care referred to the routine nursing practice of cleaning the oral cavity with antiseptic or chlorhexidine solution (30). Four key laboratory biomarkers were selected to characterize major physiological domains: SpO₂ to assess oxygenation, lactate to reflect tissue perfusion and metabolic stress, CRP to indicate systemic inflammation, and serum creatinine to represent renal function.

### Outcome

The primary outcome was 28-day all-cause mortality, determined from discharge records and verified using linked death registry data when available. The secondary outcome was in-hospital mortality, defined as death during the index hospitalization regardless of time from ICU admission. In addition, distinct biomarker trajectory classes identified during the ICU stay were treated as intermediate outcomes reflecting heterogeneous physiological response patterns, and were subsequently analyzed in relation to nursing interventions and clinical covariates.

### Statistical analysis

All analyses were performed using R software (version 4.3.1). Continuous variables are presented as mean ± standard deviation or median with interquartile range, and categorical variables as counts and percentages. Between-group comparisons for continuous variables were conducted using Student’s *t*-test or the Mann–Whitney *U* test based on data distribution, while categorical variables were compared using the *χ*^2^ test or Fisher’s exact test as appropriate.

For each biomarker, data from the first 7 days after ICU admission were used to construct longitudinal trajectories, and patients with at least three valid measurements were included. Latent class mixed-effects models were applied to identify distinct temporal trajectory patterns for SpO₂, lactate, CRP, and creatinine. Linear, quadratic, and cubic functional forms with one to five latent classes were tested. Model selection was guided by a comprehensive framework based on statistical adequacy and clinical interpretability (31, 32): (1) mean posterior probability for each class greater than 0.7; (2) class proportion exceeding 5% of the total sample; (3) clear clinical interpretability; and (4) the lowest Bayesian Information Criterion (BIC). Regarding missing data, the LCMM framework handles unbalanced longitudinal data using Full Information Maximum Likelihood (FIML) estimation under the Missing At Random (MAR) assumption. This estimation method specifically accounts for irregular measurement intervals driven by clinical indications, thereby minimizing potential bias associated with varying testing frequencies among sicker patients. This approach allows for the inclusion of all patients with at least three measurements without prior imputation.

Associations between nursing interventions and trajectory class membership were examined using multinomial logistic regression implemented with the ‘nnet’ package. Three models were constructed: Model 1 included four nursing interventions (prone positioning, EEN, airway suctioning, and oral care); Model 2 further adjusted for sex, age, and BMI; and Model 3 additionally adjusted for comorbidities including hypertension, diabetes, coronary heart disease (CHD), and chronic kidney disease (CKD). Subsequently, binary logistic regression was used to assess the associations between biomarker trajectory classes and both 28-day and in-hospital mortality, adjusting for the same covariates. All statistical tests were two-sided, and a *p*-value < 0.05 was considered statistically significant.

### Ethical considerations

This study was approved by the Ethics Committee of West China Hospital, Sichuan University (Approval No. 2025-907). The requirement for informed consent was waived due to the retrospective observational design and exclusive use of anonymized data. The reporting of this study follows the Strengthening the Reporting of Observational Studies in Epidemiology (STROBE) guidelines (33).

## Results

A total of 5,748 patients with ARDS were identified initially. After excluding non-ICU hospitalizations (*n* = 3,277), patients younger than 18 years or with missing demographic data (*n* = 49), and those with hospital stays shorter than 48 h or longer than 60 days (*n* = 280), 1,716 patients were included in the final analysis (Figure 1). Among them, 273 (15.9%) died within 28 days after diagnosis. Compared with survivors, non-survivors were older (57.6 ± 17.6 vs. 53.4 ± 16.5 years, *p* < 0.001) and had lower body mass index (23.3 ± 4.2 vs. 24.8 ± 4.7 kg/m^2^, *p* = 0.001). They also exhibited higher levels of procalcitonin and lactate and lower concentrations of serum albumin and estimated glomerular filtration rate (all *p* < 0.01), suggesting greater systemic inflammation and metabolic impairment. The distribution of ethnicity differed significantly between groups (*p* < 0.001), while sex and marital status were comparable. The prevalence of hypertension, coronary heart disease, and chronic kidney disease was similar, whereas diabetes was less frequent among non-survivors (*p* = 0.028). Differences were also noted in nursing care: non-survivors more often received airway suction and oral care (both *p* < 0.001) (Table 1).

**Table 1:** Baseline characteristics of patients with ARDS according to 28-day mortality.

Variables	28-day survivors (*n* = 1,443)	28-day non-survivors (*n* = 273)	*p*-value
Demographics			
Sex, *n* (%)			0.680
Male	973 (67.4)	180 (65.9)	
Female	470 (32.6)	93 (34.1)	
Age (mean ± SD)	53.43 (16.51)	57.58 (17.57)	<0.001
Marital status, *n* (%)			0.518
Married	1,242 (86.1)	235 (86.1)	
Unmarried	100 (6.9)	21 (7.7)	
Divorced/widowed	90 (6.2)	17 (6.2)	
Other	11 (0.8)	0 (0.0)	
Education level, *n* (%)			<0.001
Primary	240 (16.6)	25 (9.2)	
Secondary	522 (36.2)	66 (24.2)	
Higher	259 (17.9)	43 (15.8)	
Other	422 (29.2)	139 (50.9)	
Ethnicity, *n* (%)			<0.001
Han	1,017 (70.5)	132 (48.4)	
Tibetan	43 (3.0)	6 (2.2)	
Yi	13 (0.9)	0 (0.0)	
Other minorities ^a^	370 (25.6)	135 (49.5)	
Clinical characteristics			
Body mass index (mean ± SD)	24.84 (4.69)	23.32 (4.23)	0.001
White blood cell count [median, IQR]	10.90 [7.29, 15.61]	10.50 [6.66, 16.65]	0.518
C-reactive protein [median, IQR]	134.00 [75.10, 228.00]	123.00 [69.70, 217.00]	0.307
Procalcitonin [median, IQR]	1.43 [0.42, 6.90]	2.07 [0.64, 15.43]	0.001
AST/ALT ratio [median, IQR]	1.55 [1.03, 2.43]	1.72 [1.10, 2.60]	0.053
Serum creatinine [median, IQR]	85.00 [58.00, 159.00]	98.50 [63.00, 182.75]	0.008
Estimated GFR [median, IQR]	77.94 [36.23, 104.04]	55.07 [29.75, 97.65]	0.008
Alkaline phosphatase [median, IQR]	82.00 [60.00, 121.00]	82.00 [56.00, 125.75]	0.58
Direct bilirubin [median, IQR]	7.60 [4.20, 15.70]	7.00 [4.10, 14.57]	0.591
Total bilirubin [median, IQR]	14.90 [9.10, 25.50]	13.55 [8.33, 25.70]	0.212
Lactate [median, IQR]	1.90 [1.40, 2.80]	2.40 [1.70, 3.92]	<0.001
Serum albumin (mean ± SD)	30.03 (6.08)	28.89 (7.42)	0.006
Comorbidities, *n* (%)			
Hypertension	411 (28.5)	76 (27.8)	0.886
Diabetes	345 (23.9)	48 (17.6)	0.028
Coronary heart disease	29 (2.0)	9 (3.3)	0.271
Chronic kidney disease	46 (3.2)	6 (2.2)	0.495
Nursing interventions, *n* (%)			
Prone position ventilation	339 (23.5)	39 (14.3)	0.001
Early enteral nutrition	554 (38.4)	90 (33.0)	0.103
Oral care	1,109 (76.9)	181 (66.3)	<0.001
Airway suction care	838 (58.1)	195 (71.4)	<0.001

Values are expressed as mean ± SD, median [IQR], or *n* (%), as appropriate.

Continuous variables were compared using Student’s *t*-test or Mann–Whitney *U* test; categorical variables were compared using *χ*^2^ test.

a Other minorities include Hui, Manchu, Qiang, Tujia, Bai, and other ethnic groups.

### Results of trajectory modeling of key clinical indicators

**Figure 2:**
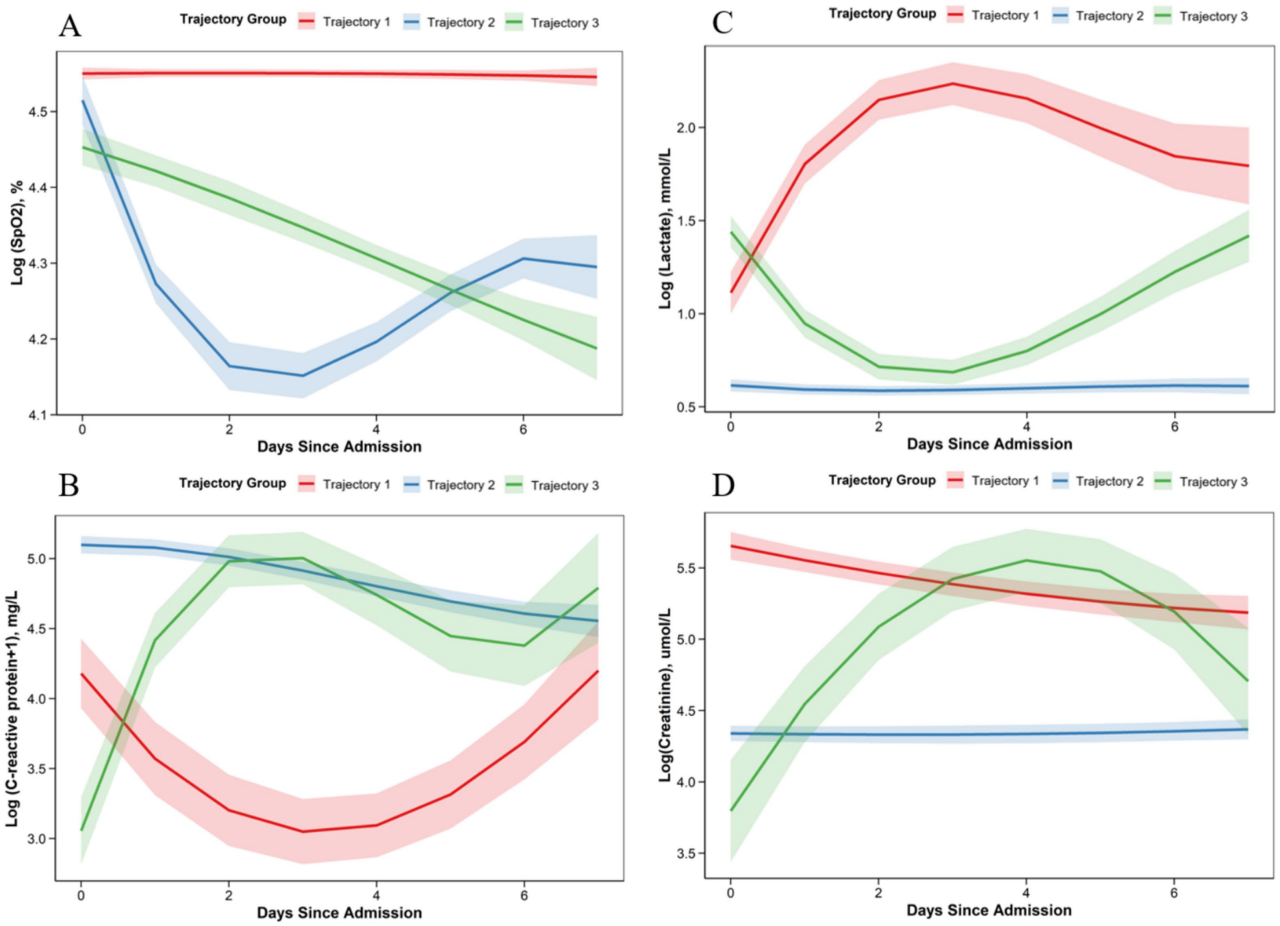
Trajectories of key physiological indicators within 7 days after ICU admission Panels **(A)**–**(D)** display the longitudinal trajectories for **(A)** SpO₂, **(B)** C-reactive protein, **(C)** lactate, and **(D)** serum creatinine. Solid colored lines represent the mean predicted trajectory for each latent class, while the semi-transparent shaded areas indicate the 95% confidence intervals. Distinct colors correspond to different subgroups: trajectory 1 (red), trajectory 2 (blue), and trajectory 3 (green). Values on the y-axes are presented on a logarithmic scale (or transformed scale) as used in the latent class mixed modeling. Abbreviations: SpO₂, peripheral oxygen saturation; CRP, C-reactive protein; ICU, intensive care unit.

Trajectory analysis was performed for four key physiological indicators within the first 7 days after ICU admission. According to model fitting indices and clinical interpretability, a three-class latent trajectory model provided the best representation for each indicator. For SpO₂, three classes reflected groups with relatively stable oxygenation (trajectory 1), a transient decline followed by recovery (trajectory 2), and a persistently declining pattern (trajectory 3) (Supplementary Table S1). For CRP, three heterogeneous trajectories were observed: a high U-shaped pattern with late re-elevation (trajectory 1), a moderate level with gradual decline (trajectory 2), and a fluctuating high-inflammation pattern (trajectory 3) (Supplementary Table S2). For lactate, trajectory 1 showed a transient rise followed by decline (inverted-U pattern), trajectory 2 remained stably low, and trajectory 3 exhibited a U-shaped profile with late re-increase (Supplementary Table S3). Similarly, three creatinine trajectories were identified, representing mildly decreasing levels (trajectory 1), relatively stable levels (trajectory 2), and a transiently increasing inverted-U pattern (trajectory 3) (Supplementary Table S4) (Figure 2).

### Association between nursing interventions and trajectory classes

**Figure 3:**
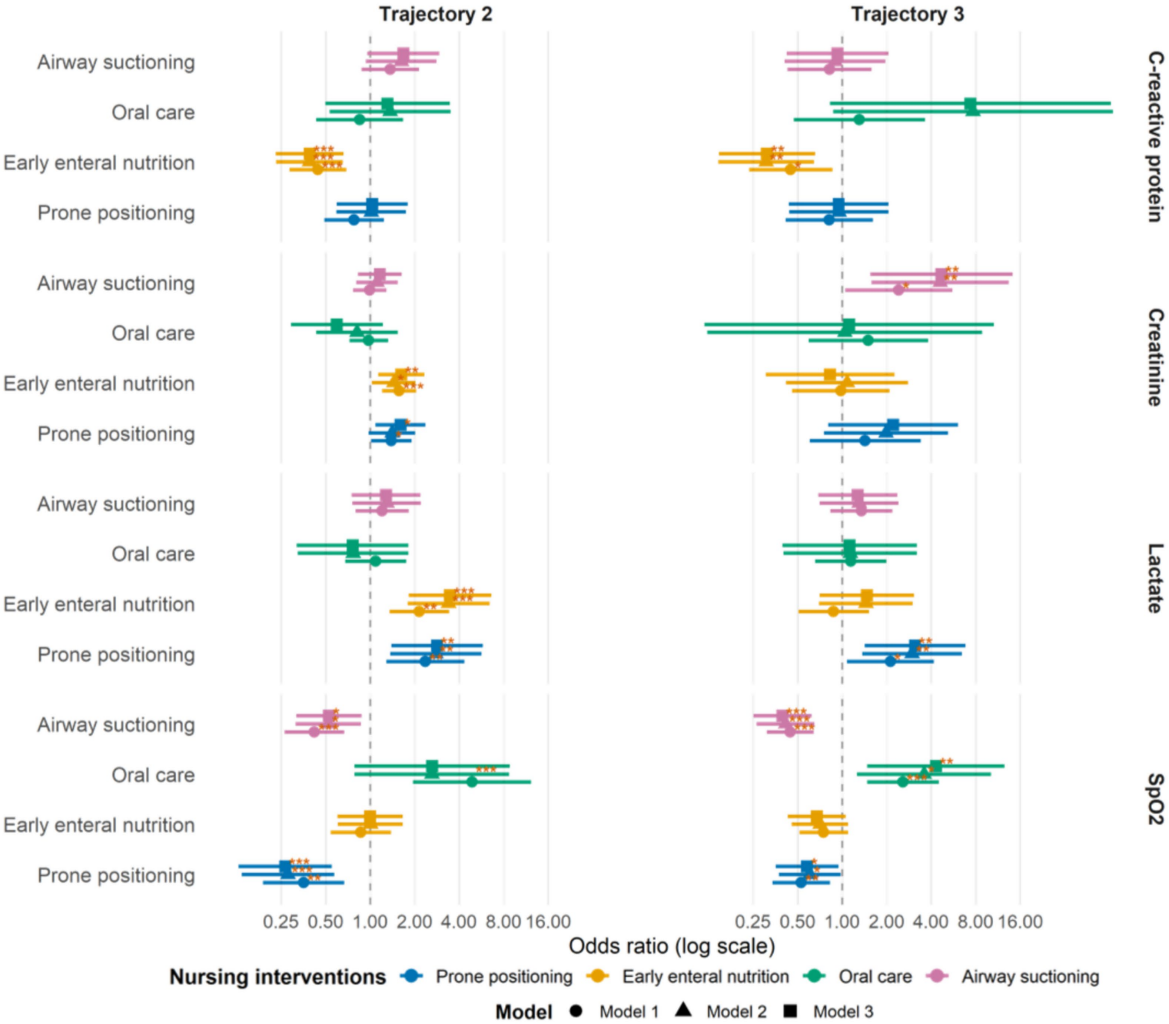
Associations between nursing interventions and biomarker trajectory classes. Forest plots illustrate the results from multinomial logistic regression models. The *x*-axis represents the OR on a logarithmic scale. Squares indicate the point estimates of the adjusted ORs, and horizontal lines represent the 95% confidence intervals. Each trajectory class (trajectory 2 and trajectory 3) was compared against trajectory 1 (the reference group). Statistical significance is denoted as: *p < 0.05, **p < 0.01, *p < 0.001.

Figure 3 presents the adjusted associations between nursing interventions and biomarker trajectory classes, using trajectory 1 as the reference. Across all three models, prone positioning was consistently associated with lower odds of unfavorable SpO₂ trajectories, including both the transient-decline–recovery pattern (trajectory 2; OR range 0.27–0.35) and the persistent-decline pattern (trajectory 3; OR range 0.53–0.60; all *p* < 0.05). Airway suction showed a similar protective association for SpO₂, with reduced odds of trajectory 2 (OR range 0.42–0.53) and trajectory 3 (OR range 0.39–0.45; all *p* ≤ 0.014). Oral care was positively associated with the unstable SpO₂ trajectory 3 across models (OR range 2.58–4.31; all *p* ≤ 0.017), whereas EEN showed no consistent association with SpO₂ trajectories. For CRP, EEN was robustly associated with lower odds of higher-inflammation trajectories in all models, including trajectory 2 (OR range 0.39–0.44) and trajectory 3 (OR range 0.31–0.45; all *p* ≤ 0.016). No consistent associations across models were observed for prone positioning, airway suction, or oral care with CRP trajectories. For lactate, both prone positioning and EEN were associated with increased odds of the stable trajectory 2 across all models (prone positioning: OR range 2.37–2.83; EEN: OR range 2.16–3.47; all *p* ≤ 0.005). Prone positioning also showed a consistent positive association with trajectory 3 (OR range 2.13–3.12; all *p* ≤ 0.029). Airway suction and oral care were not consistently associated with lactate trajectories. For creatinine, EEN was associated with increased odds of trajectory 2 across all models (OR range 1.44–1.62; all *p* ≤ 0.034). Airway suction was consistently associated with higher odds of trajectory 3 (OR range 2.42–4.70; all *p* ≤ 0.006). Other associations for creatinine were not supported across models. Detailed coefficients for all multivariable models are provided in Supplementary Table S5.

### Association of prone positioning initiation time with trajectories

In the further analysis, we found that prone positioning initiated within ≤24 h was associated with lower odds of SpO₂ trajectory 2 (OR 0.08, 95% CI 0.01–0.59; *p* = 0.013) and CRP trajectory 3 (OR 0.18, 95% CI 0.04–0.86). No other significant associations were observed in this time window. For initiation at 24–48 h, no statistically significant associations were noted across SpO₂, lactate, CRP, or creatinine trajectories, except for creatinine trajectory 2, which showed lower odds (OR 0.47, 95% CI 0.22–1.00; *p* = 0.050). For initiation at 48–72 h, no significant associations were observed for any trajectory. Prone positioning initiated >72 h was associated with higher odds of lactate trajectory 2 (OR 5.17, 95% CI 1.21–22.06; *p* = 0.027) and lactate trajectory 3 (OR 5.07, 95% CI 1.09–23.57; *p* = 0.038). It was also associated with lower odds of SpO₂ trajectory 2 (OR 0.30, 95% CI 0.09–1.00; *p* = 0.050) and SpO₂ trajectory 3 (OR 0.37, 95% CI 0.14–0.95; *p* = 0.039). For creatinine, initiation >72 h was associated with lower odds of trajectory 2 (OR 0.37, 95% CI 0.18–0.74; *p* = 0.005), while no significant association was observed for trajectory 3. The complete analysis of prone positioning timing windows is presented in Supplementary Table S6.

### Association of trajectory classes with mortality

**Figure 4:**
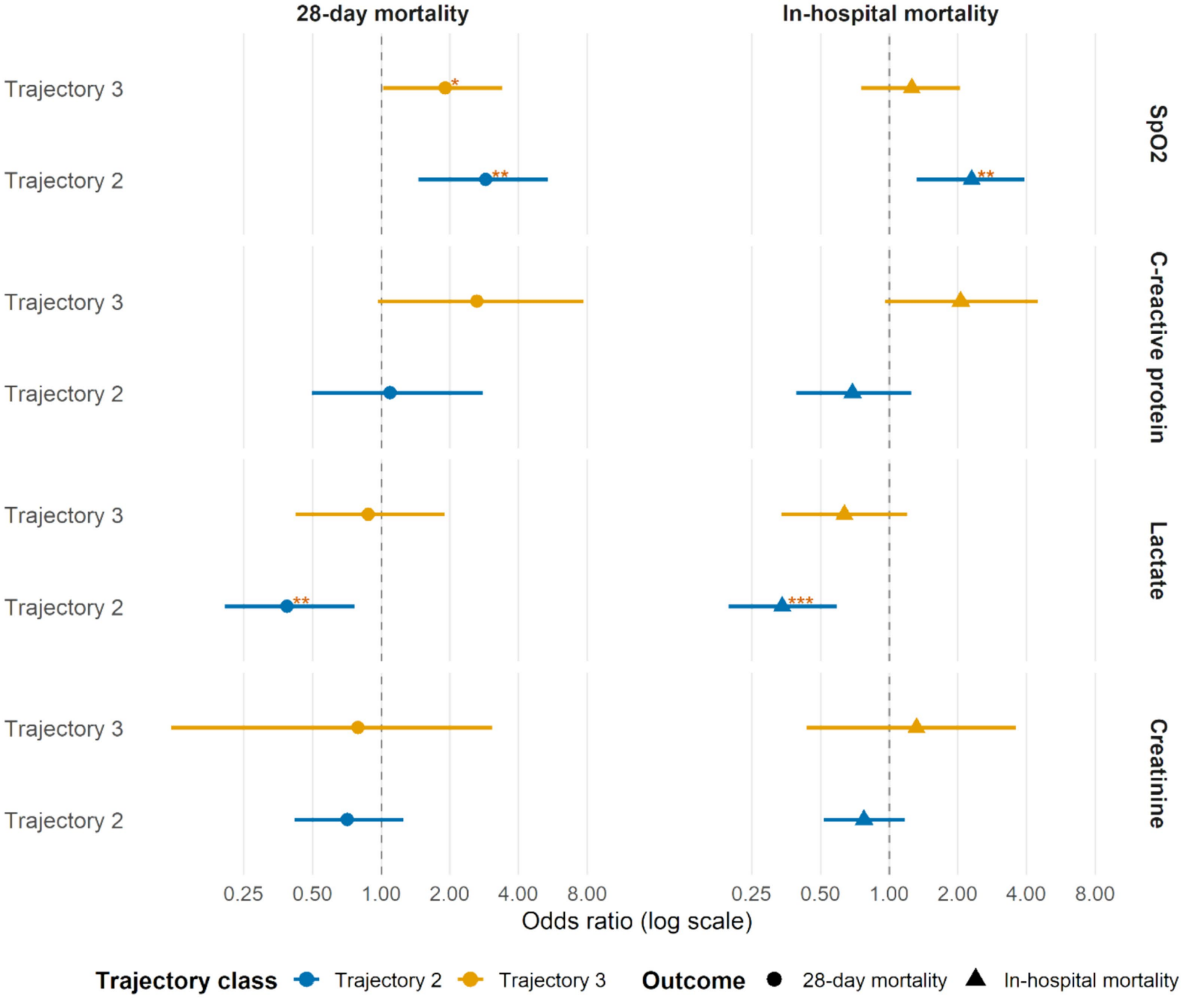
Forest plots of associations between biomarker trajectory classes and mortality outcomes. The plot displays the ORs for 28-day mortality (circles) and in-hospital mortality (triangles) associated with each trajectory class, compared to the stable/reference class (trajectory 1). Error bars indicate 95% confidence intervals.

**Figure 5:**
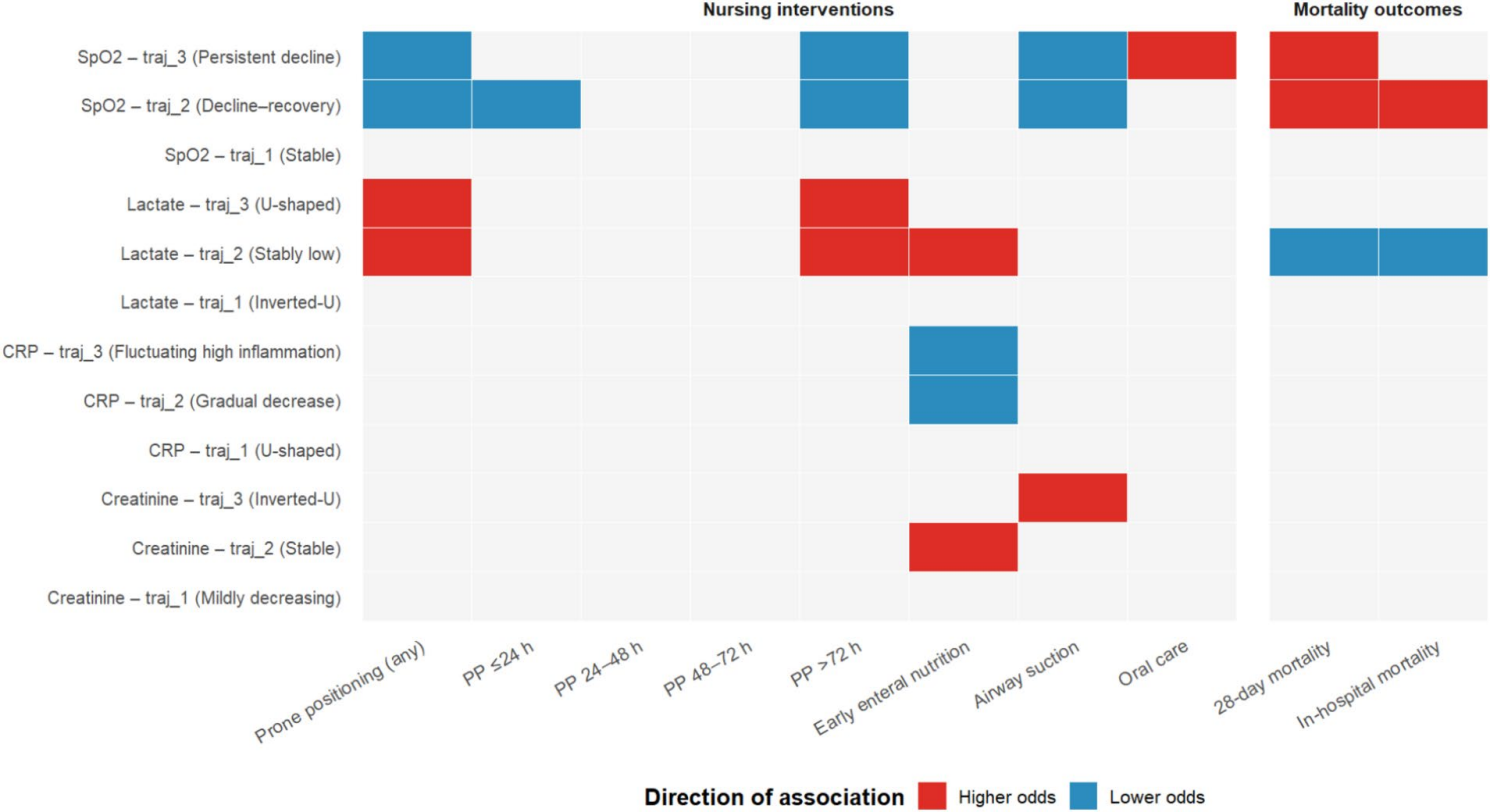
Core associations between trajectory classes, interventions, and mortality in ARDS. Rows represent biomarker trajectory classes (Reference: trajectory 1); columns indicate nursing interventions and mortality outcomes. Red cells denote higher odds (OR > 1), while blue cells denote lower odds (OR < 1). Only statistically significant associations (*p* < 0.05) are colored.

Figure 4 summarizes the associations between biomarker trajectory classes and mortality, using the stable class as the reference. For SpO₂, unstable trajectories were associated with higher risk of death. The transient-decline–recovery class (trajectory 2) was associated with greater 28-day mortality (OR 2.88, 95% CI 1.46–5.39; *p* = 0.001) and remained significant for in-hospital mortality (OR 2.30, 95% CI 1.32–3.93; *p* = 0.003). The persistent-decline class (trajectory 3) was associated with increased 28-day mortality (OR 1.91, 95% CI 1.02–3.41; *p* = 0.035) but was not associated with in-hospital mortality (OR 1.26, 95% CI 0.76–2.04; *p* = 0.365). For CRP, the higher-inflammation class (trajectory 3) showed a trend toward greater 28-day mortality (OR 2.64, 95% CI 0.96–7.72; *p* = 0.064) and in-hospital mortality (OR 2.06, 95% CI 0.96–4.48; *p* = 0.065), whereas trajectory 2 was not associated with either outcome. For lactate, the stable trajectory (trajectory 2) was associated with lower mortality, both at 28 days (OR 0.39, 95% CI 0.21–0.77; *p* = 0.004) and overall (OR 0.34, 95% CI 0.20–0.59; *p* < 0.001); trajectory 3 was not significant for either endpoint. Trajectory classes of creatinine were not significantly associated with 28-day or in-hospital mortality (see Supplementary Table S7 for full mortality analysis results). A summary of the principal results of this study is presented in Figure 5.

## Discussion

In this study, we identified distinct longitudinal trajectories for oxygenation, inflammation, metabolism, and renal function during the early course of ICU stay. Three trajectory subgroups were consistently observed for each biomarker, revealing substantial heterogeneity in physiological responses to critical illness. Early initiation of prone positioning within 24 h was linked to more favorable oxygenation and inflammatory profiles. In contrast, initiation beyond 72 h showed mixed associations with lactate and creatinine trajectories. EEN was related to lower odds of high-CRP trajectories, and airway suctioning was found to be associated with better oxygenation trajectory. In contrast, patients with persistently declining oxygenation trajectories had significantly higher short-term mortality, while elevated inflammatory trajectories were associated with a non-significant trend toward higher mortality.

Our findings significantly extend the current understanding of ARDS heterogeneity by shifting the focus from static baseline phenotypes to dynamic physiological trajectories. Prior landmark studies, particularly those by Calfee et al., have consistently identified two distinct ARDS subphenotypes, hyperinflammatory, and hypoinflammatory, based on clinical and biological data at onset (34, 35). While these static phenotypes are powerful for prognostic enrichment, they primarily reflect the severity of illness at a single time point and may not fully capture the heterogeneous responses to clinical interventions over time (36). Critical illness is inherently dynamic. A patient’s physiological state evolves rapidly in response to nursing care and therapeutic strategies. Integrating latent class mixed modeling adds a vital temporal dimension to ARDS phenotyping by disentangling heterogeneous clinical courses. For example, it distinguishes between gradual recovery and fluctuating high-inflammation patterns that may otherwise appear identical at baseline. We demonstrate that the trajectory of physiological response offers superior insight into intervention efficacy compared to static values (22). This approach advances the concept of treatable traits, shifting focus from admission syndromes to evolving phenotypes (19, 37). In comparison to prior trials of prone positioning in ARDS, our findings reinforce and extend existing evidence. Earlier meta-analyses showed that prone positioning significantly improved oxygenation and, when combined with lung-protective ventilation, reduced mortality in moderate to severe ARDS (6, 38, 39). For instance, Guérin et al. reported that daily prone positioning sessions of at least 16 h reduced 28- and 90-day mortality (6). Our results extend these findings by demonstrating that the timing of initiation is as critical as the intervention itself. Specifically, initiating prone positioning within 24 h was associated with significantly lower odds of unfavorable oxygenation trajectories. In contrast, delayed initiation (>72 h) showed mixed associations with lactate and creatinine outcomes, which likely reflects confounding by illness severity rather than a direct adverse effect.

Regarding nutritional support, our observation that EEN correlated with less inflammatory trajectory membership aligns with guideline recommendations favoring early enteral rather than parenteral feeding in critically ill patients (10, 40). For example, guidelines from ESICM/SCCM emphasize initiation of enteral nutrition within 48 h when feasible (10). Meta-analytic data suggest that early enteral feeding reduces infectious complications and may improve outcomes in heterogeneous ICU populations (40). In our ARDS cohort, EEN was linked with reduced odds of high-CRP trajectories, suggesting a plausible mechanistic link to systemic inflammation. Nevertheless, the literature also cautions that timing, patient selection, and hemodynamic stability matter, and recent cohort data did not show consistent mortality benefit solely from EEN (41). Our trajectory-based findings extend the discourse by linking timing of nutrition to evolving biomarker patterns, thereby providing additional physiological context for a care process that is widely recommended but variably implemented. Consistent with our longitudinal findings, airway suctioning appeared to be associated with more favorable oxygenation trajectories. This observation supports prior evidence that timely and adequate removal of airway secretions can improve oxygenation and reduce pulmonary inflammation in mechanically ventilated patients by preventing airway obstruction and infection (42, 43). However, we found no evidence that airway suctioning or oral care was associated with the trajectories of inflammatory markers.

ARDS is marked by diffuse alveolar–capillary barrier injury, endothelial activation, and systemic inflammation that precipitate ventilation–perfusion mismatch and multi-organ dysfunction (3, 44, 45). Within this framework, the associations observed between specific nursing interventions and biomarker trajectories suggest that early, protocolized care is linked to favorable physiological recovery at both pulmonary and systemic levels. Prone positioning improves oxygenation through multiple mechanisms, including the redistribution of transpulmonary pressures, recruitment of dorsal lung regions, and reduction of ventral overdistension. These physiological changes collectively enhance ventilation–perfusion matching and lower intrapulmonary shunt (6, 8, 46). It also reduces regional lung stress and mechanical power, mitigating ventilator-induced lung injury and the release of inflammatory mediators (7). Early initiation of prone positioning may interrupt alveolar collapse and limit endothelial activation, which is consistent with the more favorable oxygenation and inflammatory trajectories observed in our study, whereas delayed initiation showed heterogeneous associations with lactate and creatinine trajectories (6–8, 47).

EEN supports systemic recovery through maintenance of gut mucosal integrity and modulation of the immune and metabolic response (10, 12, 41, 48). It preserves splanchnic perfusion, prevents bacterial translocation, and diminishes endotoxin-driven cytokine release (10, 12). These mechanisms are consistent with our finding that EEN was associated with lower-CRP trajectories, indicating attenuation of sustained systemic inflammation. By stabilizing the gut–lung axis and improving cellular energy balance, EEN was associated with more stable lactate trajectories, indicating reduced metabolic fluctuations during the acute phase of critical illness (41). In addition, EEN was linked to the relatively stable creatinine trajectory, suggesting potential renal protection through improved perfusion and metabolic stability. Airway suctioning prevents secretion retention and maintains alveolar ventilation by reducing airway resistance and dead space (42, 43). Closed suction systems have been shown to decrease episodes of oxygen desaturation and reduce ventilator-associated complications compared with open systems (42). Our results showing more stable oxygenation trajectories among patients receiving structured suctioning are consistent with these physiological benefits and highlight the importance of maintaining airway patency during mechanical ventilation. These interventions may collectively influence organ interactions in severe ARDS. Pulmonary and endothelial inflammation can precipitate renal hypoperfusion and metabolic acidosis, while restoration of oxygenation and inflammatory trajectories reflects recovery of microcirculatory and mitochondrial function (3, 7, 44). The concurrent deterioration of lactate and creatinine trajectories seen with delayed prone positioning supports the concept that uncorrected hypoxia and inflammation accelerate lung–kidney–metabolic dysfunction.

The present study provides direct implications for clinical nursing practice in ARDS. Among all interventions examined, early prone positioning showed the most consistent association with favorable physiological trajectories. Initiation within 24 h after ICU admission was linked to rapid improvement in oxygenation and attenuation of inflammatory responses, whereas delayed implementation beyond 72 h was associated with heterogeneous lactate and creatinine trajectories. These findings reaffirm that the therapeutic window for prone positioning is narrow and emphasize the necessity of early, protocolized application to optimize gas exchange and minimize ventilator-induced lung injury (6–8). From a practical standpoint, standardized nursing checklists and early proning alerts may enhance adherence and ensure timely initiation in daily ICU workflows. Similarly, EEN was associated with reduced inflammatory trajectories, likely through preservation of intestinal barrier integrity and modulation of systemic immune responses (10, 12). This supports current guidelines advocating initiation of enteral feeding within 48 h in hemodynamically stable patients, reinforcing the critical role of nursing staff in assessing tolerance, maintaining caloric goals, and preventing gut ischemia. Airway suctioning showed an association with more favorable oxygenation trajectories, consistent with its role in maintaining airway clearance and reducing ventilation heterogeneity (42, 43). Oral care, however, was not linked to beneficial oxygenation trajectories and was more frequently provided to patients with unstable respiratory status, likely reflecting clinical response to illness severity rather than a therapeutic effect (14, 30). Consequently, the primary clinical value of our findings lies in providing mechanistic insight and a framework for risk stratification. We propose a dynamic response monitoring approach: rather than relying on static admission thresholds or waiting for a full 7-day classification, clinicians should view the failure to achieve a downward biomarker trend within the first 48 to 72 h as a critical red flag for non-response. This early identification of high-risk trajectories allows for timely escalation of care or inclusion in future adaptive clinical trials.

This study has several notable strengths that enhance both its scientific validity and clinical relevance. First, it is among the few large-scale, real-world analyses linking nursing interventions with longitudinal physiological trajectories in ARDS. By moving beyond static outcomes such as mortality, the trajectory-based framework captures the temporal evolution of oxygenation, inflammation, metabolism, and renal function, providing a dynamic way to evaluate patient heterogeneity and recovery patterns (21, 22). Second, the application of latent class trajectory modeling provided statistically and biologically coherent subgroup identification. The use of multiple biomarkers including SpO₂, lactate, CRP, and creatinine, allowed a multidimensional assessment of organ function and provided insights into the physiological pathways through which nursing interventions may interact with pathophysiology (37). Finally, by embedding detailed nursing process variables into this analytic framework, the study demonstrates the feasibility of transforming routine nursing documentation into actionable evidence that can inform data-driven nursing decisions and trajectory-informed care in ARDS management.

This study has several limitations. First, the absence of structured severity scores such as SOFA or APACHE II limits our ability to fully adjust for baseline illness severity. Although we controlled for initial physiological biomarkers, residual confounding by indication cannot be fully excluded. For instance, hemodynamically unstable patients often required stabilization before prone positioning could be safely performed, meaning delayed implementation likely reflects higher illness severity rather than treatment inefficacy. Similarly, the association between frequent airway suctioning and adverse oxygenation likely reflects underlying conditions such as excessive secretions rather than procedural harm. Second, the granularity of nursing data was limited by electronic documentation. Specifically, airway suctioning and oral care were analyzed as binary variables which precluded the assessment of technique or frequency, and our EEN data did not distinguish between trophic and full feeding targets. Third, biomarker measurement frequency was driven by clinical need rather than a fixed protocol. While our use of FIML estimation minimizes bias from irregular sampling, the trajectory shapes could still be influenced by data density in sicker patients. Fourth, as a single-center study, the findings reflect specific practice patterns in Western China, and the identified trajectory classes require validation in multi-center cohorts to ensure generalizability. Finally, given the observational design, all reported associations should be interpreted as hypothesis-generating rather than causal, and future prospective trials are required to confirm these relationships.

## Conclusion

In this large real-world cohort of patients with ARDS, distinct physiological trajectories of oxygenation, inflammation, metabolism, and renal function were identified. Early and standardized nursing interventions, particularly early prone positioning and timely enteral nutrition, were associated with more favorable recovery patterns, whereas delayed or inconsistent implementation correlated with adverse physiological trajectories. These findings suggest that high-quality, time-sensitive nursing care is significantly associated with organ recovery in ARDS. Incorporating trajectory-based monitoring into routine practice may help tailor nursing strategies and potentially improve outcomes. However, these results should be interpreted as hypothesis-generating. While they highlight the potential value of optimizing the timing of nursing care, prospective randomized controlled trials are necessary to definitively establish causal relationships between these interventions and trajectory evolution.

## Data Availability

The data analyzed in this study is subject to the following licenses/restrictions: the data supporting the findings of this study were obtained from the West China Hospital Big Data Platform. Due to the inclusion of sensitive, patient-level health information, and in accordance with the data governance policies of West China Hospital, the full de-identified dataset is not publicly available. Requests to access these datasets should be directed to Xia Li, lixia4040@163.com.
